# Seasonal regulation of condensed tannin consumption by free-ranging goats in a semi-arid savanna

**DOI:** 10.1371/journal.pone.0189626

**Published:** 2018-01-02

**Authors:** Ntuthuko R. Mkhize, Ignas M. A. Heitkӧnig, Peter F. Scogings, Dawood Hattas, Luthando E. Dziba, Herbert H. T. Prins, Willem F. de Boer

**Affiliations:** 1 Resource Ecology Group, Wageningen University, Wageningen, the Netherlands; 2 Agricultural Research Council, Animal Production Institute, Hilton, South Africa; 3 School of Life Sciences, University of KwaZulu-Natal, Pietermaritzburg Campus, Scottsville, South Africa; 4 Department of Biological Sciences, University of Cape Town, H.W. Pearson building, Rondebosch, Cape Town, South Africa; 5 Council for Scientific and Industrial Research, Natural Resources and the Environment, Pretoria, South Africa; 6 School of Life Sciences, University of KwaZulu-Natal, Westville Campus, Durban, South Africa; Institute of Zoology, CHINA

## Abstract

Although condensed tannins (CTs) are known to reduce forage intake by mammalian herbivores in controlled experiments, few studies have tested these effects in the field. Thus the role of CTs on foraging ecology of free-ranging herbivores is inadequately understood. To investigate the effects of CTs under natural savanna conditions, we pre-dosed groups of goats with polyethylene glycol (PEG, a CT-neutralising chemical), CT powder or water before observing their foraging behaviour. While accounting for the effects of season and time of the day, we tested the hypothesis that herbivores forage in ways that reduce the intake rate (g DM per minute) of CTs. We expected pre-dosing goats with CTs to reduce CT intake rates by (1) consuming diets low in CTs, (2) reducing bite rates, (3) increasing the number of foraging bouts, or (4) reducing the length of foraging bouts. Lastly, (5) expected CT to have no influence the number of dietary forage species. In both wet and dry seasons, pre-dosing goats with CTs resulted in lower CT consumption rates compared to PEG goats which seemed relieved from the stress associated with CT consumption. During dry season, the number of dietary forage species was similar across treatments, although goats that were dosed with PEG significantly increased this number in the wet season. Dosing goats with PEG increased the number and length of browsing bouts compared to goats from the other treatments. Pre-loading goats with PEG also tended to increase bite rates on browse forages, which contributed to increased consumption rates of CTs. Based on the behavioural adjustments made by goats in this study and within the constraints imposed by chemical complexity in savanna systems, we concluded that herbivores under natural conditions foraged in ways that minimised CTs consumption. More research should further elucidate the mechanism through which CTs regulated feeding behaviour.

## Introduction

Condensed tannins (CTs) are widely distributed among the nutritionally important forages in the African savanna rangelands [1,2], and their consumption by mixed feeding and browsing herbivores is unavoidable [3,4,5]. To reduce the deleterious effects of dietary CTs, herbivores are purported to regulate their daily intake of browse species such that CT intake is minimized without compromising the overall dry matter intake [6,7]. Although intake regulation of CTs is expected to involve altering of meal patterns, these short-term alterations are not clearly understood. Feeding experiments show captive herbivores to reduce the intake rate (g DM per minute) of forages containing compounds with known anti-nutritional, toxic or digestibility-reducing effects [8,9].

The length of feeding bouts has been reported to be shorter for animals exposed to toxin-containing forage sources than those exposed to toxin-free forages [8,10,11]. Marsh et al. [9] reported high concentrations of formylated phloroglucinol compounds to cause koalas to eat more slowly, eat shorter meals and eat less per meal. Moreover, Wiggins et al [6] indicated that plant secondary metabolites not only constrain overall intake, but also alter feeding behaviour of the animals. Altered feeding patterns are believed to reduce the negative influence of PSMs on intake [12,13]. However, we know of no rigorous tests of these short-term behavioural alterations over the timescale of feeding bouts and inter-bout intervals with regard to CTs [8]. Given that rumen microbes are not capable of degrading CTs [14,15], these compounds are unlikely to be absorbed and transported to liver cells, and therefore may not induce foraging alterations similarly to the PSMs (i.e. toxins) that are detoxified through activation of liver enzymes [16]. Therefore, CTs are different from toxins and are expected to affect foraging behaviour differently.

Forages that are rich in CTs likely require longer chewing and digestive processing time than similar forages that contain little or no CTs [17]. Thorough chewing by herbivores with CT-binding salivary proteins [18,19,20] is required to facilitate effective insalivation of food during mastication, which may lower intake rates (g DM per minute) through reducing the bite rates [8]. Moreover, in the presence of CTs in the rumen (pH 5.5–7.0), most of the dietary proteins and carbohydrates remain bound and protected from microbial degradation [21]. However, some of the bound protein are released later in the abomasum (pH 2.5 to 5.1) enabling protein digestion and amino acid absorption in the small intestines [22]. We therefore, hypothesized that herbivores foraging in African savanna rangelands that are dominated by CT-rich woody plants would forage in ways that reduce their intake rates (g DM per minute) of CTs.

Mixed feeders are known to consume varied diets (i.e. mixed diets) as a means to maintain high forage intake from plants that are rich in different secondary metabolites while avoiding excessive ingestion of individual plant secondary metabolites [23]. This has been explained in terms of the detoxification limitation hypothesis [24] which predicts varied diets to spread detoxification of toxins over many metabolic pathways, thereby reducing constraints on liver enzymes and substrates [25]. While this hypothesis has been tested mostly on toxins [9,26,27], the extent to which CTs influence the number of dietary species and diet composition in the field is poorly understood. Savanna rangelands are complex systems characterised by many plant species. These plants are likely defended by a complex and diverse myriad of chemical compounds [28] which makes it almost unlikely to completely isolate the effects of CTs from other tannins and phenolic compounds especially in the field [29,30]. However, while savanna plants use various chemicals to defend against herbivory, CTs have been extensively reported as the most abundant chemical defence against mammalian herbivores in the African savannas [31,32,33,34]. Given that CTs are not toxic and thus are not detoxified via the liver, we would not expect goats that are foraging in CT-rich environments to increase dietary species diversity. Instead we predicted goats in the African savannas to deal with CT constraint by switching their diets from CT-rich to CT-poor forages.

To determine the effects of CTs on short-term foraging behaviour and diet composition of free-ranging goats in a semi-arid African savanna, we pre-dosed goats with (1) polyethylene glycol (PEG, an anti-tannin agent dissolved in water), (2) CT powder dissolved in water or (3) only water. While accounting for the effects of season and time of the day, we tested the hypothesis that free-ranging goats in the African savannas forage in ways that reduce the intake rate of CTs. To some extent, these treatments attempted to change animal exposure to CTs while leaving exposure to other compounds unaltered. We predicted that pre-dosing goats with CTs will lead to (a) maintain the number of dietary forage species, (b) consumption of diets lower in CTs (c) reduction in bite rates, (d) increase in number of foraging bouts, or (e) reduction in foraging bout length. We defined a bout as a period of continuous foraging on a particular forage species separated by either a non-foraging activity [35] or by foraging on a different plant species. We used goats as an important model organism for understanding feeding behaviour of mixed feeders, such as impala, lamas, steenbok, deer or eland [36,37].

## Materials and methods

### Study area

We carried out a field experiment at the Roodeplaat Experimental Farm located in Pretoria, South Africa (25°20´-25°40´E; 28°17´-28°25´S). The climate is semi-arid with a mean annual rainfall of 646 mm and mean daily maximum temperatures between 20–29°C in January and 2–16°C in July [38]. The main wet season occurs from November to April, and the dry season starts in May and reaches its peak in July. The vegetation of the farm falls within the savanna biome and is classified as Marikana Thornveld [39]. The rangeland is dominated by *Acacia karroo*, *Acacia tortilis*, *Ziziphus mucronata* and some *Euclea* species. Nomenclature of plants followed Coates Palgrave [40].

### Study design

Forty five (45) indigenous yearling female goats ranging from 8 to 12 months old with an initial body weight of 14.9 (standard deviation ± 3.7) kg were used in this experiment. All study goats were sourced from the experimental farm of the Agricultural Research Council in Pretoria and they were allocated to three treatment groups such that all groups had an equal number of goats (N = 15) and a similar mean body weight. Fifteen goats received a daily oral dose with 20g of polyethylene glycol (PEG 6000) dissolved in 50 ml of water whereas another 15 were dosed with 50 ml of water plus 20 g of CT extract (from mimosa bark) (MIMOSA Extract Company (Pty) Ltd., Pietermaritzburg, South Africa) and the last 15 received 50 ml of water (control) before they were released to the field. The mimosa extract was obtained from the bark of the Black Wattle (*Acacia mearnsii*) tree and contained a minimum of 66% CTs on dry matter basis. Three grazing camps/paddocks of similar size (1.8 ha) were fenced and stocked with 15 goats (i.e., 5 from each treatment group) daily from 08:00 until 16:00. All study goats were treated for internal and external parasites before the experiment and had *ad libitum* access to water throughout the experiment. From 08:00 onwards, all goats were allowed to forage freely in the field until 16:00 when they were corralled to avoid predation. The goats were corralled 1.2 km away from the camps and they received free-access to water and no feed while in the corral. The experiment was approved by the Animal Ethics Committee of the ARC under permit number: APIEC11/039.

### Data collection

The foraging behaviour of goats in the field was recorded during the dry (June-August 2012) and wet (January-March 2013) seasons. To aid easy identification during observations, we marked all study goats with paint on the flanks. To habituate the goats to the presence of observers and to allow close monitoring of behaviour, we subjected them to a two week conditioning period before the actual observations. On each day of the actual observation, nine goats (i.e., 3 from each treatment group) were randomly selected and observed. The three goats observed per treatment group would be foraging in different paddocks. Of the nine goats observed each day, three (one from each treatment group) were observed in the early morning (08:00 to 10:30), three observed in the late morning (10:30 to 12:00) and the other three observed in the afternoon (12:00 to 15:30).

One observer followed each goat for fifteen minutes, while assisted by one recorder throughout the experiment. The same team observed and recorded the foraging behaviour throughout the experiment. Per observation, we identified and counted the number of dietary forage species, and recorded the date, starting time, treatment, paddock and the goat number for each observation. The observations were conducted for thirty days and thus a total of 270 observations lasting 15 min each (i.e., 9 observations per day x 30 days) were made per season. We used the Observer XT 10.5 [41] in combination with a Psion Work-about handheld computer [41,42] to record the forage species being eaten, bite rate (number of bites per minute during foraging), number of times a goat foraged from each species (number of bouts) and the amount of time (seconds) the goat spent on each foraging bout (bout length). Grazing was recorded as one species (i.e., “grass”) without distinguishing the different species.

To estimate the tannin concentration [CT] of the diet consumed by the study goats, we sampled leaves of all plant species included by goats in the diet, and analysed them for CTs. In each season, a minimum of 8 leaf samples (each with fresh weight of 20g) were collected from unbrowsed branches at about 1.2 m height or lower. Collected leaves were oven-dried at 60 ^o^C till completely dry. Dried samples were finely ground to pass through a 1mm screen and stored in plastic honey jars pending chemical analysis. Condensed tannins were determined using the acid-butanol assay method [43]. Since it was not possible to purify all forage species consumed by goats, a purified sorghum was used a standard for CT estimation [44]. This analysis allowed us to estimate the [CTs] (mg/g equivalent on dry matter basis) for each plant species, which we further report as [CTs] (mg/g). Sampling was carried out during dry and wet season separately and the lab analyses were conducted at the Botany laboratory of the Department of Biological Sciences, University of Cape Town.

To estimate the amount of CTs consumed (g DM) per minute, we multiplied the total number of bites taken from each plant species by the mean [CT] (mg/g DM) and by the mean bite size (g DM) for each plant species in each season. Since it was not possible to estimate bite sizes from the field, we, estimated the bite size in a pen experiment. Bite size was not only important in estimating the amount of food (g DM) per bite (which was used in estimating CT intake), but it was also important in estimating intake rates achievable from each forage species. During each of the two seasons (one day after the field observations), we selected 10 of the study goats and penned them individually under a shelter. At least 10 un-browsed branches of each of the plant species that were included by goats in the diet during field observations, were collected from the sites in which the field observations were done. We then estimated the bite sizes (g DM per bite) according to Mkhize et al. [45].

For each plant species we estimated the average proportion inclusion in the diet of each goat. This percentage was calculated as the quotient of the consumption (g DM) of each plant species and total consumption of all species during an observation multiplied by 100.

### Data analysis

Differences in (1) CT intake rate, (2) bite rates, (3) length of feeding bouts and (4) intake (g DM) by goats from different treatment groups were analysed with linear models using a manual backward selection of variables. In each model, season (dry and wet), treatment (PEG, CT and control) and time of day (early morning, late morning and afternoon) were fixed factors, while the paddock served as a random factor. The unstandardized residuals of CT intake rates model were normally distributed only after a natural log transformation. All other response variables met the normality and variance homogeneity assumptions without any transformation. A generalized linear model, with a Poisson distribution was used to analyse the effect of season, treatment and time of the day on (1) the number of forage species in the diet and (2) the number of foraging bouts. We applied a Sidak test for pairwise comparisons between different treatment groups, seasons and times of the day. We also conducted a simple regression analysis of CT content and consumption. All analyses were performed using SPSS, v20 (IBM SPSS Statistics; Chicago, IL, USA).

## Results

**Fig 1:**
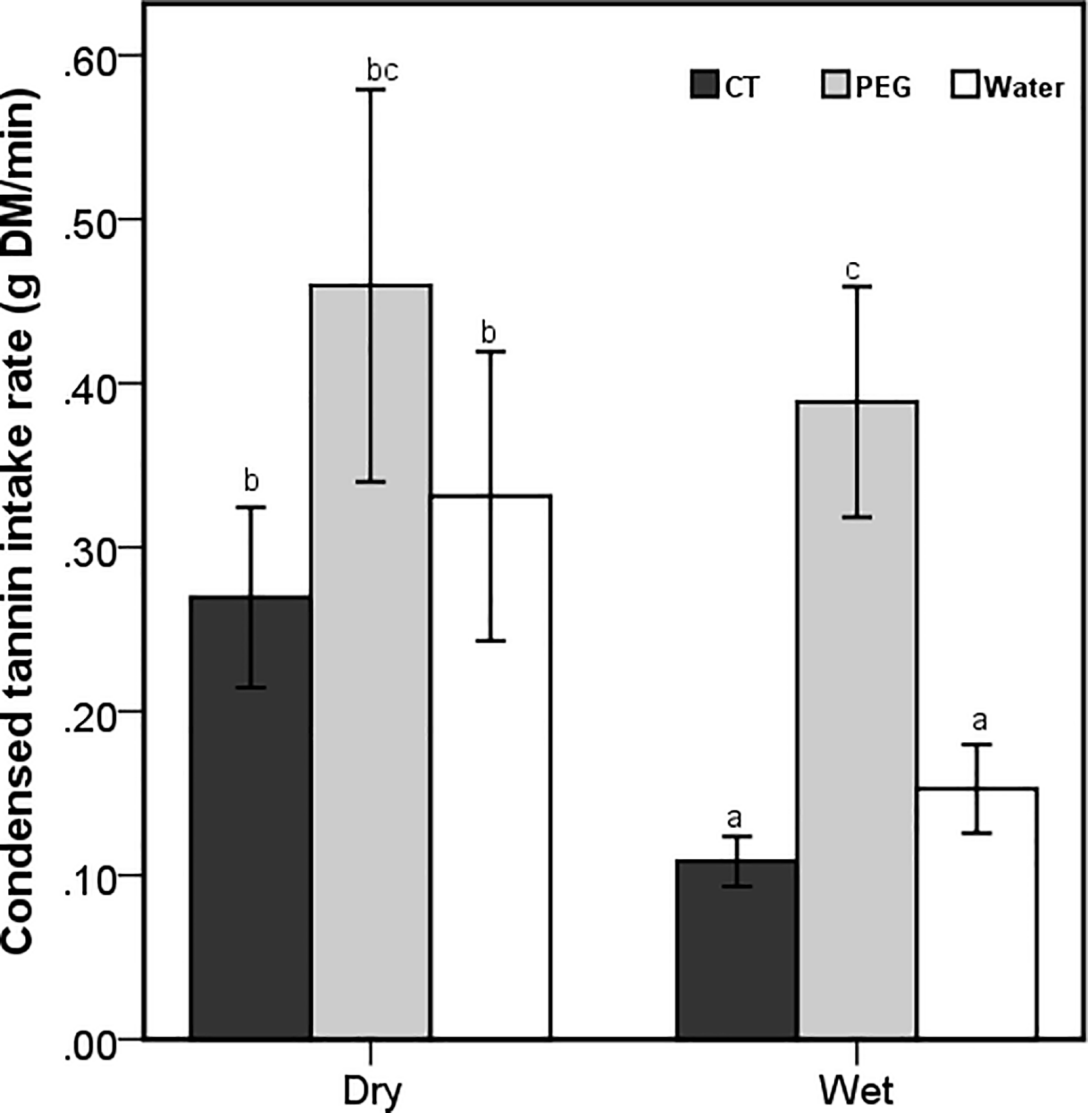
Mean (±95% CI) condensed tannin intake rate (g DM per minute foraging) of free-ranging goats that were orally dosed with 20g of condensed tannins (CT), 20g of polyethylene glycol (PEG) and 50ml of water daily during the dry and wet season. Letters represent significant differences among seasons and treatments.

There was a significant interaction between treatment and season on CT intake rates of free-ranging goats (F_2,533_ = 11.69; *P* < 0.001; Fig 1). All goats, except for those that were dosed with PEG, achieved lower CT intake rates in the wet than in the dry season (Fig 1). In both seasons, control and CT-dosed goats tended to consume CTs at lower rates than the PEG-dosed goats. Although further analysis showed a gradual decline in CT intake rates from early-morning to the afternoon across all treatments, the time of the day did not significantly influence CT intake rates.

**Fig 2:**
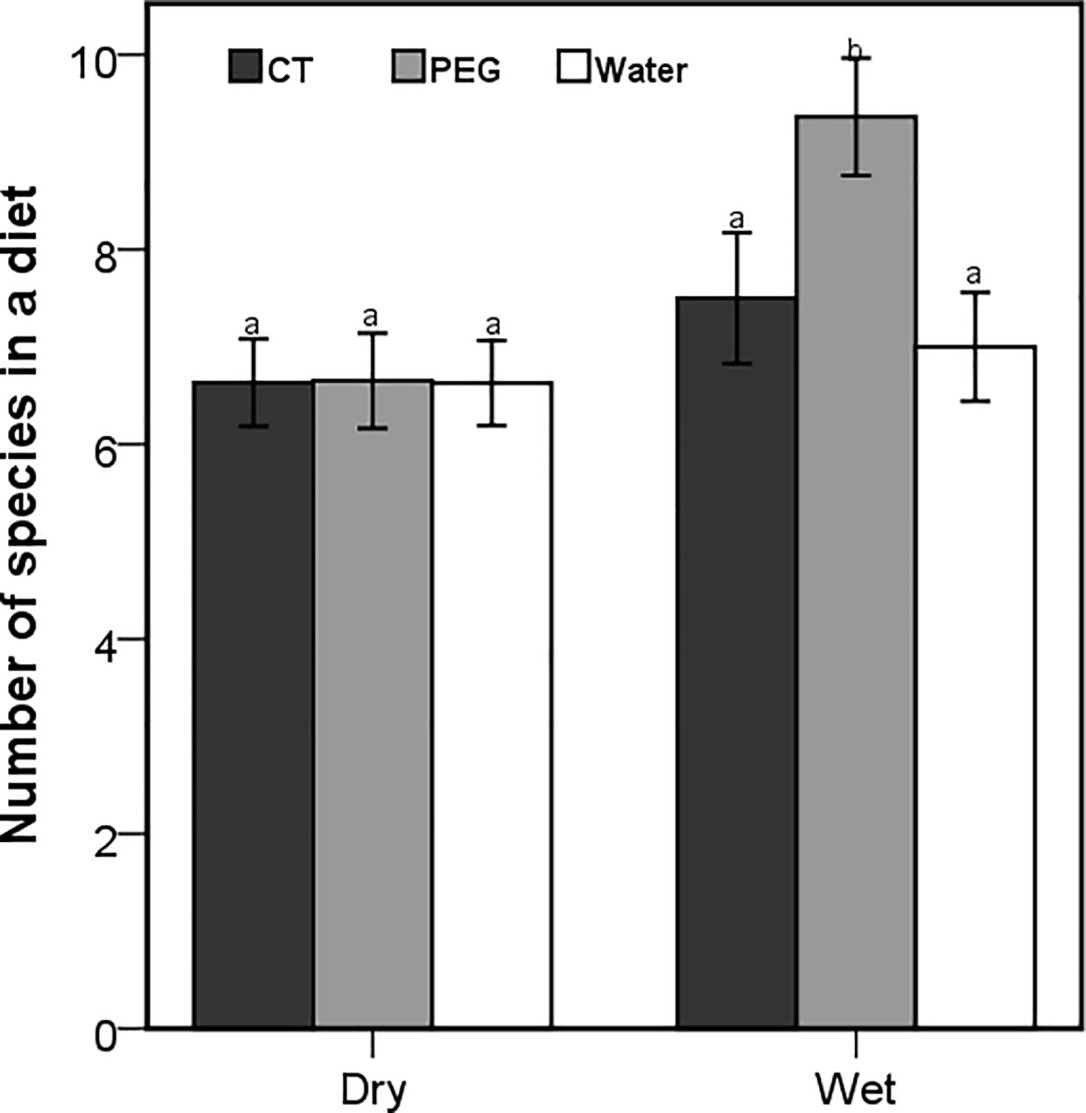
The mean (±95% CI) number of forage species included by free-ranging goats in their diets during a 15 minutes observation. The goats were orally dosed with 20g of condensed tannins (CT), 20g of polyethylene glycol (PEG) and 50ml of water daily during the dry and wet seasons. Letters represent significant differences among seasons and treatments.

The number of forage species included by goats from all treatment groups was the same during the dry season and slightly, but not significantly increased for CTs and control goats in wet season. Dosing goats with PEG significantly increased the number of dietary plant species included in the diet during the wet season (Wald X^2^ = 15.53; *P* < 0.001, Fig 2). Goats dosed with PEG consumed more browse and relatively less grass than the other treatment groups. All browse species were eaten less by the control goats and those pre-dosed with CTs than goats dosed with PEG, independently of the [CT] in the dietary plants (F_2,141_ = 4.83; *P* = 0.009). The [CT] of browse species did not have any relationship with the percentage contribution of the species in the diet (R^2^ = 0.035; *P* = 0.380, Table 1).

**Table 1:** Condensed tannin (CT) composition (mg/g) of forage plants and average % consumption (g DM) for each plant species consumed by goats dosed with CT, PEG and water per observation during dry and wet seasons.

	CT composition	Dry season (% g DM intake in the diet)			CT composition	Wet season (% g DM intake in the diet)		
Forage Species	mg/g (N)	CT-group	PEG-group	Water-group	mg/g (N)	CT-group	PEG-group	Water-group
*Acacia caffra*	149.93 (6)	1.59	11.16	2.50	141.71 (6)	4.86	6.00	7.19
*Acacia karroo*	103.06 (9)	0.85	5.70	2.07	133.95 (6)	2.35	6.09	7.65
*Acacia nilotica*	4.94 (8)	1.59	5.92	1.18	18.82 (5)	0.95	2.58	0.57
*Acacia robusta*	16.27 (10)	1.92	4.77	1.80	13.33 (6)	2.31	5.40	6.02
*Acacia tortilis*	21.90 (4)	0.65	2.07	0.06	51.25 (6)	0.76	2.64	1.92
*Aloe greatheadii*	0.70 (1)	5.56	3.63	3.48	2.60 (3)	3.95	2.50	3.48
*Berchemia zeyheri*	28.19 (1)	10.66	12.34	9.16	54.26 (3)	1.21	10.69	5.29
*Carissa bispinosa*	95.57 (10)	1.22	2.86	2.08	111.73 (5)	0.41	2.41	0.43
*Combretum apiculatum*	50.42 (8)	30.52	43.50	31.60	56.10 (4)	32.62	59.08	0.00
*Combretum zeyheri*	17.54 (6)	3.24	13.03	0.35	26.43 (6)	4.21	23.11	1.68
*Dichrostachys cinerea*	38.61 (5)	0.42	18.08	2.48	75.39 (6)	2.93	7.50	11.36
*Dombeya rotindifolia*	52.81 (9)	12.11	16.36	9.00	62.25 (6)	10.10	26.87	51.66
*Ehretia rigida*	1.40 (9)	7.61	8.45	5.50	2.25 (6)	4.20	8.91	6.78
*Euclea crispa*	69.02 (12)	6.11	7.78	10.38	70.49 (6)	2.46	10.40	8.39
Grass		57.99	33.91	54.37		66.37	22.09	55.90
*Grewia flava*	48.56 (5)	0.53	0.80	0.56	67.03 (6)	3.80	7.29	5.09
*Gymnosporia buxifolia*	68.92 (9)	3.04	12.36	2.57	68.57 (6)	3.10	9.90	4.89
*Pappea capensis*	48.46 (11)	7.13	9.81	4.22	78.46 (6)	2.59	4.05	3.85
*Rhus lancea*	129.35 (9)	18.27	19.35	27.64	28.73 (6)	9.08	20.69	17.65
*Rhus Leptodictya*	175.69 (14)	5.21	1.32	1.80	76.91 (6)	0.84	4.87	2.55
*Rhus pyroides*	63.49 (2)	0.35	0.75	0.23	93.57 (4)	6.98	10.09	8.40
*Scolopia zeyheri*	72.79 (7)	0.68	1.45	1.19	83.64 (4)	1.17	2.06	0.90
Herbs	7.07 (7)	4.58	5.35	5.18	6.30 (5)	4.28	6.97	5.22
*Ziziphus mucronata*	40.51 (10)	12.20	25.08	17.03	41.87 (6)	5.81	11.40	9.33

**Fig 3:**
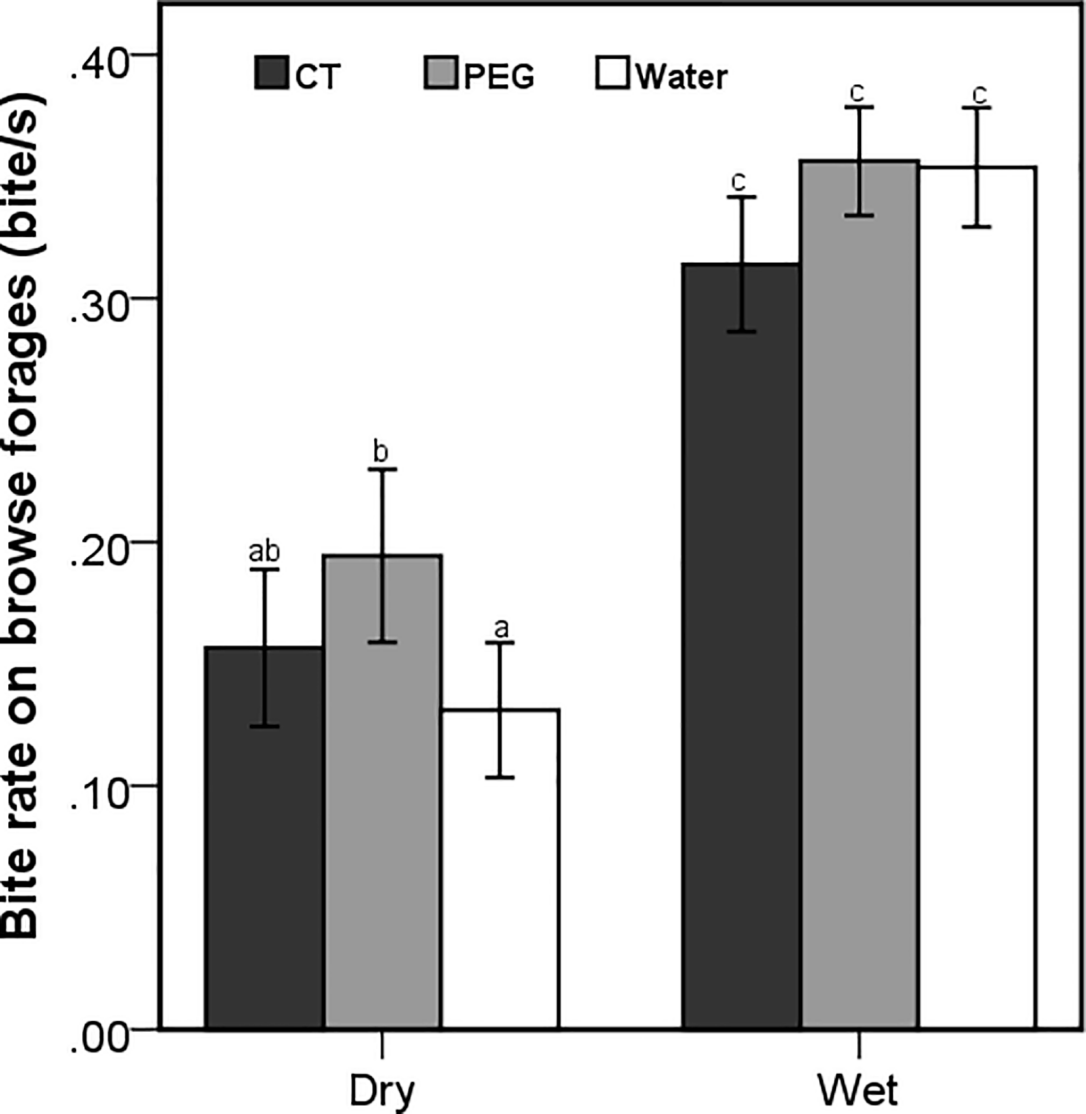
Mean (±95% CI) bite rates while browsing on woody plants by free-ranging goats that were orally dosed with 20g of condensed tannins (CT), 20g of polyethylene glycol (PEG) and 50ml of water daily during the dry and wet season. Letters indicate significant differences among the seasons and treatments.

The distinctive nature of biting by goats when grazing and browsing necessitated separate bite rate analysis for browsing and grazing. Bite rate on browse forages was significantly influenced by the interaction between season and treatment (F_2,517_ = 3.15; *P* = 0.044; Fig 3). The bite rates achieved by goats across the treatment groups were consistently lower in the dry season and almost doubled in the wet season (Fig 3). Interestingly, the bite rates while grazing were only influenced by season (F_1,530_ = 8.75; *P* = 0.003), with significantly higher rates in the dry than in the wet season.

**Fig 4:**
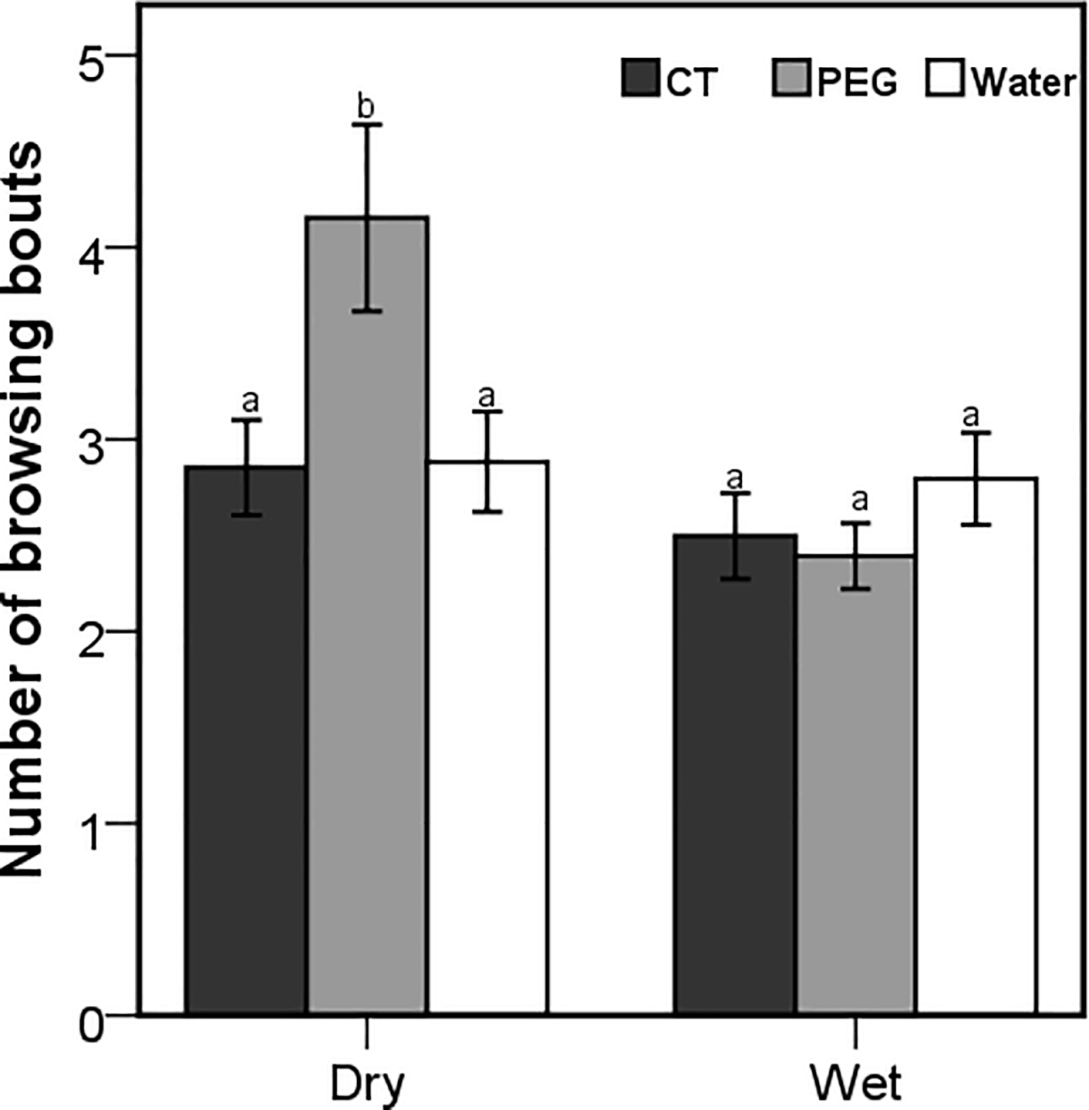
Mean (±95% CI) number of browsing bouts during a 15 minute observation of free-ranging goats that were orally dosed with 20g of condensed tannins (CT), 20g of polyethylene glycol (PEG) and 50ml of water daily in dry and wet seasons. Letters indicate significantly different means among the seasons and treatments.

**Fig 5:**
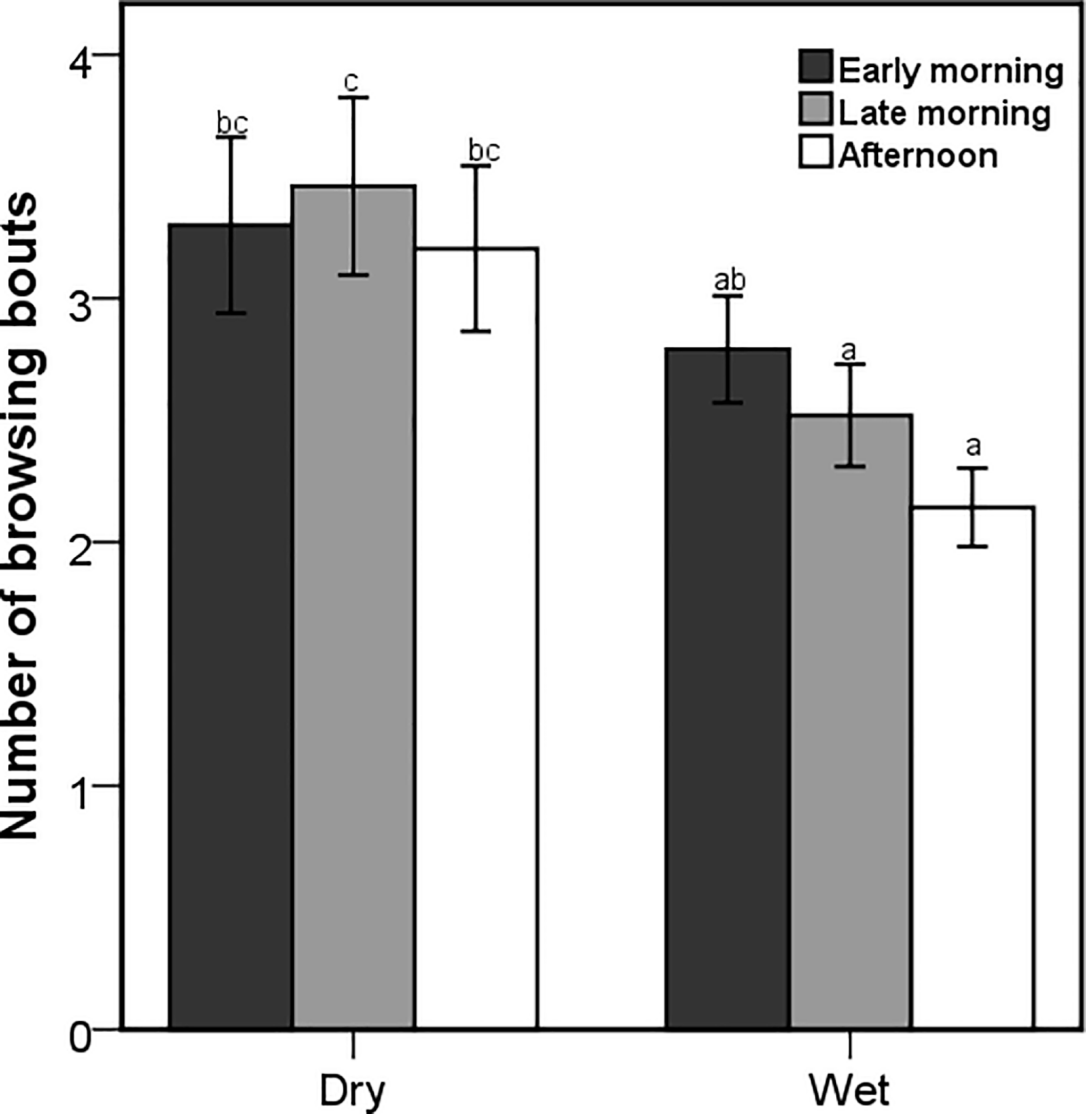
Mean (±95% CI) number of browsing bouts during a 15 minute observation of free-ranging goats in the wet and dry season and in early morning, late morning and afternoon. Letters indicate significantly different means among the seasons and treatments.

**Fig 6:**
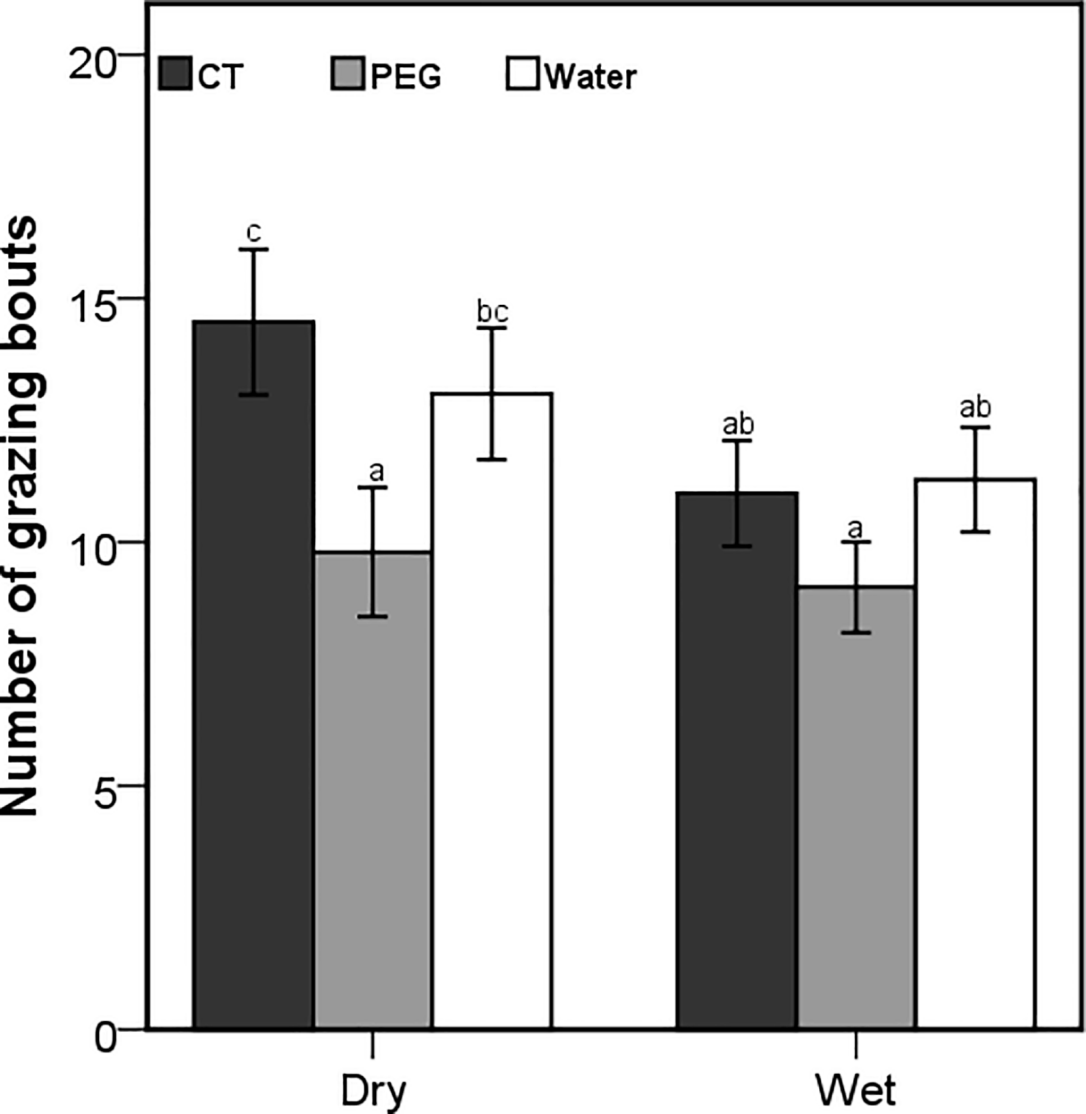
Mean (±95% CI) number of grazing bouts per 15 minute observation period for the free-ranging goats that were orally dosed with 20g of condensed tannins (CT), 20g of polyethylene glycol (PEG) and 50ml of water daily during the dry and wet seasons. Letters represent significant differences among seasons and treatments.

**Fig 7:**
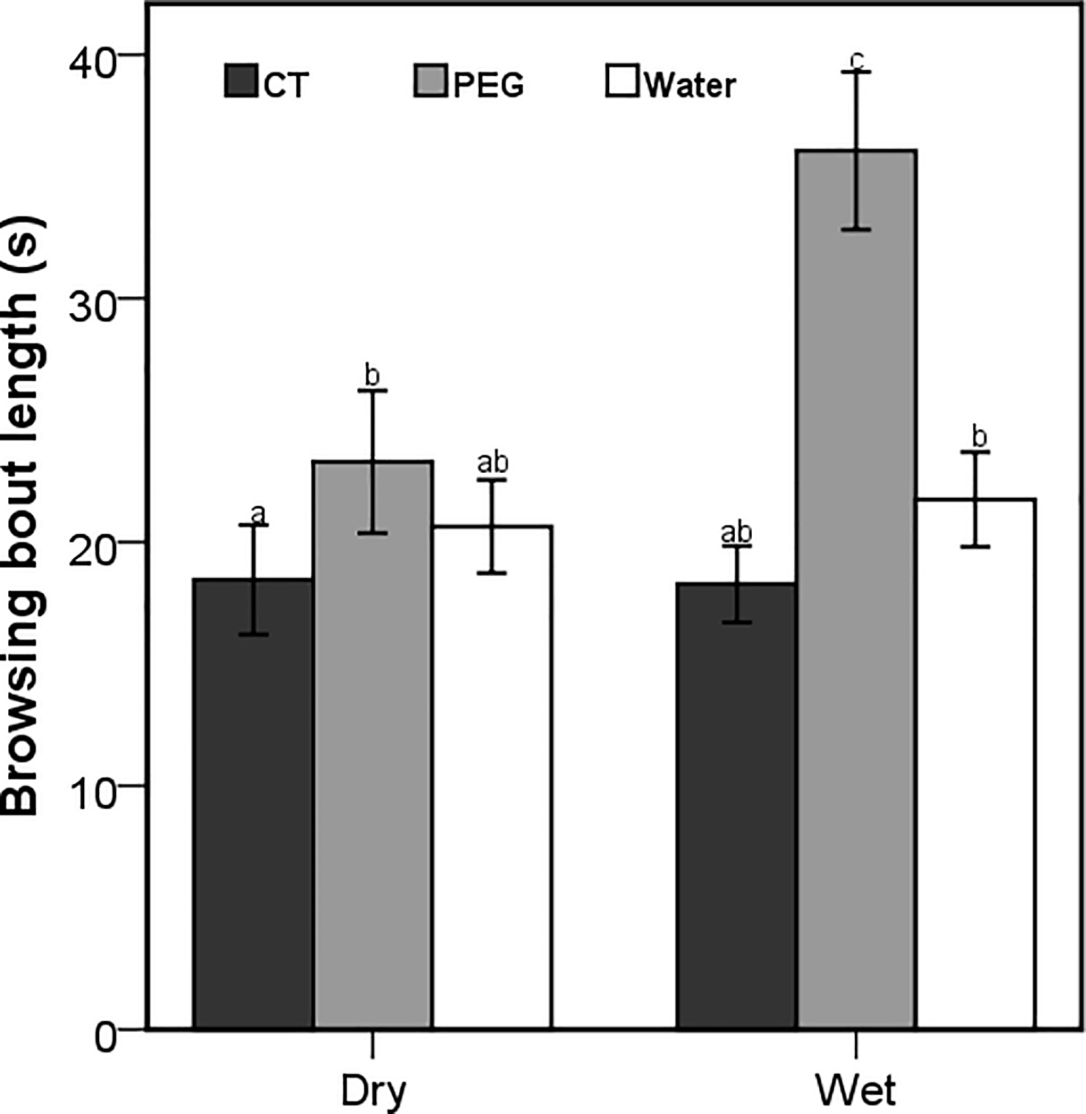
Mean (±95% CI) browsing bout length (s) for the free-ranging goats orally dosed with 20g of condensed tannins (CT) 20g of polyethylene glycol (PEG) and 50ml of water daily during the dry and wet seasons. Letters represent significant differences among seasons and treatments.

**Fig 8:**
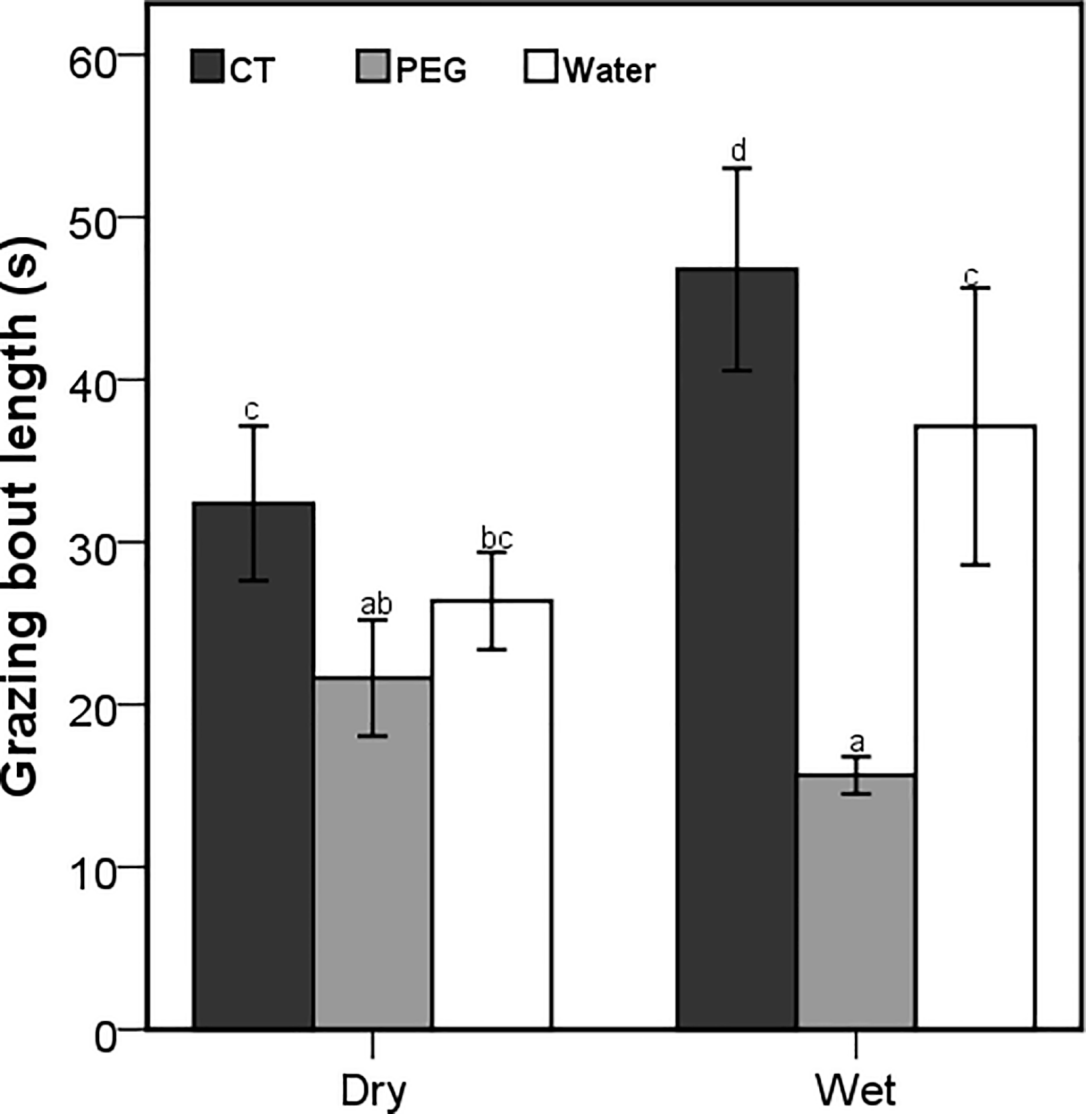
Mean (±95% CI) grazing bout length (s) for the free-ranging goats orally dosed with 20g of condensed tannins (CT) 20g of polyethylene glycol (PEG) and 50ml of water daily during the dry and wet seasons. Letters represent significant differences among seasons and treatments.

Although the number of browsing bouts was affected by the interaction between season and treatment (Wald X^2^ = 171.47; *P* < 0.001), only the goats that were dosed with PEG during dry season had a significantly higher number of browsing bouts (Fig 4). The number of browsing bouts was also influenced by the interaction between the season and time of the day (Wald X^2^ = 19.42; *P* < 0.001), with significant differences during the late morning and afternoon foraging periods (Fig 5). The season x treatment interaction also influenced the number of grazing bouts (Wald X^2^ = 10.17; *P* = 0.006), with the CT-goats recording the highest and the PEG goats recording the lowest number of grazing bouts in both seasons (Fig 6). Browsing bout length differed significantly among seasons and treatments (F_2,3414_ = 7.81; *P* < 0.001), with the goats treated with PEG achieving the longest and CT-dosed goats achieving the shortest bouts in both seasons (Fig 7). The opposite was found for the grazing bout length (Fig 8). which was also affected by the interaction between season and treatment (F_2,530_ = 8.09; *P* < 0.001).

## Discussion

We tested the hypothesis that the foraging behaviour of free-ranging goats enables them to regulate intake rates of CTs in the African savannas. Condensed tannins are well known to influence foraging behaviour in controlled feeding experiments [46,47]. As predicted, goats that were pre-dosed with CTs achieved lower intake rates of CTs than the PEG group. Reduced consumption rates of CTs by CT-dosed and control goats were likely a physiological strategy to contend with the adverse effects of CTs on nutrient metabolism [48].

PEG is potentially a powerful anti-CT agent in the context of animal nutrition [10], and was used in this study to experimentally create different levels of CT stress among the study goats. While PEG has widely been used indiscriminately to bind CTs in similar studies, it is still not clear whether other tannins and phenolic compounds are not also bound by this compound [30]. Dosing goats with CTs constrained their CT intake rate, PEG goats seemed to be relieved from this CT constrain. PEG is known to preferentially bind CTs, thereby reducing the CTs’ detrimental protein-binding effects [49,50,51]. Our results suggest a limited ability of goats to reduce CT intake rate in the dry season, and we found higher amounts and rates of CT intake during dry season. This was in line with findings of other studies [52,53]. One explanation for this is the loss of leaves among deciduous trees during the dry season. Goats have fewer options and have to optimally utilize those available options to meet their nutritional needs. One coping strategy, which is discussed in detail later, is to reduce the length, while increasing the number, of feeding bouts during the dry season.

Cautious sampling and diet mixing by herbivores have been assumed to generally increase the intake of forages that are rich in secondary metabolites [7,54]. The current results based on CTs did not support this notion owing to the plant secondary metabolites in question being only digestibility reducers and not toxins. In explaining these results it is important to note that no data were available on whole spectrum of the bioactive chemicals in the study area which may serve as a limitation for ecological studies that measure presence of one tannin (CTs) in a system where other types of tannins such as hydrolysable tannins [30,55], ellagitannins [29] and other phenolic compounds may also be present [28]. Pre-dosing goats with CTs or PEG did not influence dietary botanical diversity of goats. However, the results show the number of dietary species to be consistently higher for all treatments in the wet than in the dry season. Season may have influenced the number of dietary forages through reducing browse forage availability in dry season. This is a common phenomenon for the seasonal semi-arid African savannas [56,57]. Deciduous species lose leaves during dry season, thus limiting the forage options for mixed feeders and browsing herbivores to a few evergreen woody species and grass. Dosing goats with CTs in the wet season forced them to focus their foraging on grass [58]. It also increased the grazing bout length and the number of grazing bouts at the expense of browsing bouts. This may indicate the existence of a threshold for CT intake above which a higher dietary species diversity does not benefit herbivores. Hence the number of dietary species was similar between the CT-dosed goats and the control goats. The high species diversity among PEG dosed goats may suggest that goats were more tolerant for CT rich forages which enabled them to include more species in their diet. On the other hand, pre-dosing goats with CTs forced goats to select and consume a diet that is lower in CTs in the field.

Herbivores can modify their intake patterns as an important strategy to minimize consumption of plant secondary metabolites [59]. Specific behavioural modifications that goats use to regulate intake of plant secondary metabolites include adjusting their total intake, intake rate, length and/or number of bouts, length between feeding bouts, or to switch their diet composition [6,7,12,59]. We predicted pre-dosing goats with CTs to increase the number of foraging bouts while at the same reducing the length of foraging bouts, as a means to regulate intake rates of CTs. Bite rates appeared to be influenced more by the season than by treatment. We also predicted that pre-dosing goats with CTs would reduce bite rates, thereby reducing their intake rates, especially intake rates of CT rich species. A clear difference between the goats dosed with PEG and those from the other two groups indicated some potential effects of CTs on browse bout and browse bout length. For example, PEG goats achieved the highest number of bouts during the dry season, the season in which they achieved the highest CT intake rates. The control group and the CT-dosed goats achieved less frequent bouts in the dry season compared to PEG dosed goats possibly due to them achieving CT satiation levels sooner than their PEG counterparts. This supports previous studies that have shown CTs to limit diet intake although this study is further demonstrating the effects under field conditions. There were no differences in number of feeding bouts during the wet season. The higher number of grazing bouts by CT-dosed and control goats shows that these goats preferred to graze than browsing, possibly due to higher CT content in browse compared to grass (see Table 1). The consistent similarities in terms of bite rates, bout number and bout length between CTs and control groups may suggest a similar CT stress level between these two groups. This may therefore indicate that our CTs treatment was not as effective in stressing foraging behaviour as anticipated, or that the control goats were already heavily CT-stressed. Our results were obtained from free-ranging goats, which allowed the goats to make a broader choice from the available forage species than those in previous studies in pens [27]. Previous studies on the effects of plant secondary metabolites on meal patterns were done with captive animals, which may have had fewer behavioural options than free-ranging animals. Although our study did not show CTs to significantly reduce bite rates as we expected, the results indicated that CTs do not only constrain overall intake of CT-rich plants but they also alter their short-term foraging behaviour.

These results support our hypothesis that the foraging behaviour of free-ranging herbivores in the African savannas enables them to control intake rates of CTs. Although more work is still needed to further explain the mechanisms governing CT-intake regulation, this study demonstrates that pre-dosing herbivores with CTs reduced their consumption rate of woody plants (CT-containing forages) in favour of grass. We interpret this reduction in CT intake rate as the need for herbivores to regulate CT intake [9] in an effort to decrease the digestibility-reducing effects. We demonstrated that pre-dosing herbivores with CTs leads to significant alterations by animals in numbers of foraging bouts and length of foraging bouts. We explained the observed foraging alterations as means to regulate intake rates of CTs. Although pre-dosing goats with CTs did not reduce bite rates, reduced bout length or increased the number of foraging bouts, pre-dosing with PEG evidently decreased CT-stress in the field. Thus we concluded that herbivores under natural conditions alter their bite rate, bout number and bout length in ways that regulate CT consumption. However, given that tannins are highly variable in structure and tannin composition varies even individuals and organs of the same plant species [8,29], more research is needed to provide ecologists and chemists alike with alternative methods that allow holistic investigation of important research questions such those addressed in this paper.

## Supporting information

S1 Data Data analysed during the experiment showing independent variables that include season, paddock, treatment and time of the day and various dependent variables. (SAV)

## References

[1] Mueller-Harvey I (2006) Unravelling the conundrum of tannins in animal nutrition and health. Journal of the Science of Food and Agriculture 86: 2010–2037.

[2] Barbehenn RV, Constabel CP (2011) Tannins in plant-herbivore interactions. Phytochemistry 72: 1551–1565. doi: 10.1016/j.phytochem.2011.01.040 21354580 10.1016/j.phytochem.2011.01.040

[3] Provenza FD (1996) Acquired aversions as the basis for varied diets of ruminants foraging on rangelands. Journal of Animal Science 74: 2010–2020. 8856457 10.2527/1996.7482010x

[4] Sorensen JS, Heward E, Dearing MD (2005) Plant secondary metabolites alter the feeding patterns of a mammalian herbivore (Neotoma lepida). Oecologia 146: 415–422. doi: 10.1007/s00442-005-0236-8 16163555 10.1007/s00442-005-0236-8

[5] Sorensen JS, McLister JD, Dearing MD (2005) Novel plant secondary metabolites impact dietary specialists more than generalists (Neotoma spp.). Ecology 86: 140–154.

[6] Wiggins NL, McArthur C, McLean S, Boyle R (2003) Effects of two plant secondary metabolites, cineole and gallic acid, on nightly feeding patterns of the common brushtail possum. Journal of Chemical Ecology 29: 1447–1464. 12918927 10.1023/a:1024221705354

[7] Marsh KJ, Wallis IR, McLean S, Sorensen JS, Foley WJ (2006) Conflicting demands on detoxification pathways influence how common brushtail possums choose their diets. Ecology 87: 2103–2112. 16937649 10.1890/0012-9658(2006)87[2103:cdodpi]2.0.co;2

[8] Foley WJ, Iason GR, McArthur C (1999) The role of plant secondary metabolites in the nutritional ecology of mammalian herbivores: how far have we come in 25 years? In Nutritional Ecology of Herbivores: 130–209.

[9] Marsh KJ, Wallis IR, Foley WJ (2007) Behavioural contributions to the regulated intake of plant secondary metabolites in koalas. Oecologia 154: 283–290. doi: 10.1007/s00442-007-0828-6 17690913 10.1007/s00442-007-0828-6

[10] Foley W, Iason G, Makkar H. Transdisciplinary studies of plant secondary metabolites: Lessons from ecology for animal science and vice versa; 2007.

[11] Dearing MD, Foley WJ, McLean S (2005) The influence of plant secondary metabolites on the nutritional ecology of herbivorous terrestrial vertebrates. Annual Review of Ecology Evolution and Systematics 36: 169–189.

[12] Wiggins NL, McArthur C, Davies NW (2006) Diet switching in a generalist mammalian folivore: fundamental to maximising intake. Oecologia 147: 650–657. doi: 10.1007/s00442-005-0305-z 16328546 10.1007/s00442-005-0305-z

[13] Wiggins NL, Marsh KJ, Wallis IR, Foley WJ, McArthur C (2006) Sideroxylonal in *Eucalyptus* foliage influences foraging behaviour of an arboreal folivore. Oecologia 147: 272–279. doi: 10.1007/s00442-005-0268-0 16205948 10.1007/s00442-005-0268-0

[14] Makkar HPS, Becker K, Abel H, Szegletti C (1995) Degradation of Condensed Tannins by Rumen Microbes Exposed to Quebracho Tannins (Qt) in Rumen Simulation Technique (Rusitec) and Effects of Qt on Fermentative Processes in the Rusitec. Journal of the Science of Food and Agriculture 69: 495–500.

[15] Makkar HPS, Blummel M, Becker K (1995) In-Vitro Effects of and Interactions between Tannins and Saponins and Fate of Tannins in the Rumen. Journal of the Science of Food and Agriculture 69: 481–493.

[16] Makkar HPS (2003) Effects and fate of tannins in ruminant animals, adaptation to tannins, and strategies to overcome detrimental effects of feeding tannin-rich feeds. Small ruminant research 49: 241–256.

[17] Terrill T, Waghorn GC, Woolley D, McNabb W, Barry T (1994) Assay and digestion of C-labelled condensed tannins in the gastrointestinal tract of sheep. British Journal of Nutrition 72: 467–477. 7947660 10.1079/bjn19940048

[18] Hanovice-Ziony M, Gollop N, Landau SY, Ungar ED, Muklada H, et al (2010) No major role for binding by salivary proteins as a defense against dietary tannins in Mediterranean goats. Journal of Chemical Ecology 36: 736–743. doi: 10.1007/s10886-010-9809-z 20559693 10.1007/s10886-010-9809-z

[19] Shimada T (2006) Salivary proteins as a defense against dietary tannins. Journal of Chemical Ecology 32: 1149–1163. doi: 10.1007/s10886-006-9077-0 16770710 10.1007/s10886-006-9077-0

[20] Juntheikki MR, JulkunenTiitto R, Hagerman AE (1996) Salivary tannin-binding proteins in root vole (*Microtus oeconomus* Pallas). Biochemical Systematics and Ecology 24: 25–+.

[21] Hagerman AE, Robbins CT, Weerasuriya Y, Wilson TC, Mcarthur C (1992) Tannin chemistry in relation to digestion. Journal of Range Management 45: 57–62.

[22] Barry TN, Manley TR (1984) The role of condensed tannins in the nutritional-value of *Lotus-Pedunculatus* for Sheep .2. Quantitative digestion of garbohydrates and proteins. British Journal of Nutrition 51: 493–504. 6722090 10.1079/bjn19840055

[23] Bailey DW, Provenza FD (2008) Mechanisms determining large-herbivore distribution. Resource Ecology: 7–28.

[24] Freeland WJ, Janzen DH (1974) Strategies in herbivory by hammals—role of plant secondary compounds. American Naturalist 108: 269–289.

[25] Marsh KJ, Wallis IR, Andrew RL, Foley WJ (2006) The detoxification limitation hypothesis: Where did it come from and where is it going? Journal of Chemical Ecology 32: 1247–1266. doi: 10.1007/s10886-006-9082-3 16770716 10.1007/s10886-006-9082-3

[26] Marsh KJ, Wallis IR, Foley WJ (2005) Detoxification rates constrain feeding in common brushtail possums (*Trichosurus vulpecula*). Ecology 86: 2946–2954.

[27] Dearing MD, Mangione AM, Karasov WH (2000) Diet breadth of mammalian herbivores: nutrient versus detoxification constraints. 123: 397–405. doi: 10.1007/s004420051027 28308595 10.1007/s004420051027

[28] Hattas D, Hjalten J, Julkunen-Tiitto R, Scogings PF, Rooke T (2011) Differential phenolic profiles in six African savanna woody species in relation to antiherbivore defense. Phytochemistry 72: 1796–1803. doi: 10.1016/j.phytochem.2011.05.007 21621803 10.1016/j.phytochem.2011.05.007

[29] Salminen J-P, Karonen M (2011) Chemical ecology of tannins and other phenolics: we need a change in approach. Functional Ecology 25: 325–338.

[30] Rautio P, Bergvall UA, Karonen M, Salminen JP (2007) Bitter problems in ecological feeding experiments: commercial tannin preparations and common methods for tannin quantifications. Biochemical Systematics and Ecology 35: 257–262.

[31] Sittenauer JM, Fischer JK, Dahm ME, D'Silva CL, Grant LM, et al (2013) Characterization of hydrolyzable tannins in the leaf galls of *Quercus palustris* and investigation of their ecological significance. Abstracts of Papers of the American Chemical Society 245.

[32] Chapman GA, Bork EW, Donkor NT, Hudson RJ (2010) Effects of supplemental dietary tannins on the performance of white-tailed deer (*Odocoileus virginianus*). Journal of Animal Physiology and Animal Nutrition 94: 65–73. doi: 10.1111/j.1439-0396.2008.00883.x 19364384 10.1111/j.1439-0396.2008.00883.x

[33] Scogings PF, Hjalten J, Skarpe C (2011) Secondary metabolites and nutrients of woody plants in relation to browsing intensity in African savannas. Oecologia 167: 1063–1073. doi: 10.1007/s00442-011-2042-9 21660581 10.1007/s00442-011-2042-9

[34] Owen-Smith N (1993) Woody plants, browsers and tannins in southern African savannas. South African Journal of Science 89: 505–510.

[35] De Castro JM (1975) Meal pattern correlations: facts and artifacts. Physiology & behavior 15: 13–15. 1197393 10.1016/0031-9384(75)90271-1

[36] McNaughton SJ, Georgiadis NJ (1986) Ecology of African grazing and browsing mammals. Annual Review of Ecology and Systematics 17: 39–65.

[37] Hofmann RR (1989) Evolutionary steps of ecophysiological adaptation and diversification of ruminants: a comparative view of their digestive system. 78: 443–457. doi: 10.1007/BF00378733 28312172 10.1007/BF00378733

[38] Panagos MD, Westfall RH, van Staden JM, Zacharias PJK (1998) The plant communities of the Roodeplaat Experimental Farm, Gauteng, South Africa and the importance of classification verification. South African Journal of Botany 64: 44–61.

[39] Mucina L, Rutherford MC (2006) The vegetation of South Africa, Lesotho and Swaziland. Pretoria: South African National Biodiversity Institute viii, 807 p. p.

[40] Coates Palgrave K (2002) Trees of southern Africa. New edition revised and updated by Meg Coates Palgrave Cape Town: Struik 1212: 118.

[41] Noldus L (1991) The Observer: a software system for collection and analysis of observational data. Behavior Research Methods, Instruments, & Computers 23: 415–429. 10.3758/bf03195516 14587547

[42] Zimmerman P, Bolhuis JE, Willemsen A, Meyer E, Noldus LJJ (2009) The Observer XT: A tool for the integration and synchronization of multimodal signals. Behavior Research Methods 41: 731–735. doi: 10.3758/BRM.41.3.731 19587185 10.3758/BRM.41.3.731

[43] Porter LJ, Hrstich LN, Chan BG (1986) The Conversion of procyanidins and prodelphinidins to cyanidin and delphinidin. Phytochemistry 25: 223–230.

[44] Hattas D, Julkunen-Tiitto R (2012) The quantification of condensed tannins in African savanna tree species. Phytochemistry Letters 5: 329–334.

[45] Mkhize NR, Scogings PF, Dziba LE, Nsahlai IV (2011) Season and plant species influence foraging efficiency of Nguni goats in pens. African Journal of Range & Forage Science 28: 29–34.

[46] Min BR, Barry TN, Attwood GT, McNabb WC (2003) The effect of condensed tannins on the nutrition and health of ruminants fed fresh temperate forages: a review. Animal Feed Science and Technology 106: 3–19.

[47] Waghorn G (2008) Beneficial and detrimental effects of dietary condensed tannins for sustainable sheep and goat production-Progress and challenges. Animal Feed Science and Technology 147: 116–139.

[48] Villalba JJ, Provenza FD, Banner RE (2002) Influence of macronutrients and activated charcoal on intake of sagebrush by sheep and goats. Journal of Animal Science 80: 2099–2109. 12211378 10.2527/2002.8082099x

[49] Rogosic J, Pfister JA, Provenza FD, Pavlicevic J (2008) The effect of polyethylene glycol on intake of Mediterranean shrubs by sheep and goats. Journal of Animal Science 86: 3491–3496. doi: 10.2527/jas.2007-0828 18765854 10.2527/jas.2007-0828

[50] Decandia A, Cabiddu A, Sitzia A, Molle G (2008) Polyethylene glycol influences feeding behaviour of dairy goats browsing on bushland with different herbage cover. Livestock Science 116: 183–190.

[51] Villalba JJ, Provenza FD (2002) Polyethylene glycol influences selection of foraging location by sheep consuming quebracho tannin'. Journal of Animal Science 80: 1846–1851. 12162651 10.2527/2002.8071846x

[52] Jansen DA, van Langevelde F, de Boer WF, Kirkman KP (2007) Optimisation or satiation, testing diet selection rules in goats. Small Ruminant Research 73: 160–168.

[53] Nyamangara ME, Ndlovu LR (1995) Feeding-behavior, feed-intake, chemical and botanical composition of the diet of indigenous goats raised on natural vegetation in a semiarid region of Zimbabwe. Journal of Agricultural Science 124: 455–461.

[54] McLean S, Duncan AJ (2006) Pharmacological perspectives on the detoxification of plant secondary metabolites: implications for ingestive behavior of herbivores. Journal of Chemical Ecology 32: 1213–1228. doi: 10.1007/s10886-006-9081-4 16770714 10.1007/s10886-006-9081-4

[55] Moilanen J, Salminen J-P (2008) Ecologically neglected tannins and their biologically relevant activity: chemical structures of plant ellagitannins reveal their in vitro oxidative activity at high pH. Chemoecology 18: 73–83.

[56] Owen-Smith N, Cooper SM (1985) Comparative consumption of vegetation components by kudus, impalas and goats in relation to their commercial potential as browsers in savanna regions. South African Journal of Science 81: 72–76.

[57] Bryant JP, Kuropat PJ, Cooper SM, Frisby K, Owen-Smith N (1989) Resource availability hypothesis of plant antiherbivore defense tested in a South-African savanna ecosystem. Nature 340: 227–229.

[58] Mkhize NR, Heitkönig IMA, Scogings PF, Dziba LE, Prins HHT, et al (2015) Condensed tannins reduce browsing and increase grazing time of free-ranging goats in semi-arid savannas. Applied Animal Behaviour Science 169: 33–37.

[59] Estell RE (2010) Coping with shrub secondary metabolites by ruminants. Small Ruminant Research 94: 1–9.

